# Typical Case of Converter Smelting with High Cooling Ratio in Chinese Iron and Steel Enterprises: CO_2_ Emission Analysis

**DOI:** 10.3390/ma18010065

**Published:** 2024-12-27

**Authors:** Huapeng Yang, Chao Feng, Yubin Li, Feihong Guo, Rong Zhu, Minke Zhang, Xing Wang, Xin Du, Liyun Huo, Fuxin Wen, Tao Ren, Guangsheng Wei, Fuhai Liu

**Affiliations:** 1 Institute for Carbon Neutrality, University of Science and Technology Beijing, Beijing 100083, China; y18810725270@163.com (H.Y.); zhurongtzh@163.com (R.Z.); wei18810725270@sina.com (G.W.); 2 Institute of Steel Sustainable Technology, Liaoning Academy of Materials, Shenyang 110000, China; 3 Sinosteel Equipment & Engineering Co., Ltd., Beijing 100083, China; liyubinsteel@126.com (Y.L.); guofeihongiron@126.com (F.G.); zhangminke2@aliyun.com (M.Z.); huoliyunsteel@aliyun.com (L.H.); 4 Sinosteel Engineering & Technology Co., Ltd., Beijing 100083, China; 5 Shandong Iron and Steel Group Rizhao Co., Ltd., Rizhao 276500, China; wangxing2@aliyun.com (X.W.); wen18500797366@sina.com (F.W.); 6 Panshi Jianlong Steel Co., Ltd., Panshi 132300, China; duxinsteel@126.com; 7 Rizhao Steel Holding Group Co., Ltd., Rizhao 276500, China; rentaoiron@139.com; 8 National Center for Materials Service Safety, University of Science and Technology Beijing, Beijing 100083, China

**Keywords:** converter, CO_2_ emissions, high scrap ratio, heating agent, biochar

## Abstract

In this study, the effects of using different scrap ratios in a converter on carbon emissions were analyzed based on life cycle assessment (LCA) theory, and the carbon emissions from the converter were evaluated with the use of coke and biochar as heating agents at high scrap ratios. In this industrial experiment, the CO_2_ emissions during the converter smelting process decreased with the increase in the scrap steel ratio. For every 1% increase in the scrap steel ratio, the carbon emissions during the steelmaking process decreased by 14.09 kgCO_2_/t steel. Based on statistical data for the actual use of a charcoal heating agent in the converter, the relationship between the utilization coefficient of the heating agent and the scrap ratio was calculated as $\eta =7.698\times 10^{2}x^{-2.596}.$ When biochar was used as a converter heating agent, the scrap ratio required to achieve the lowest carbon emissions was 36%, and the converter emissions could be reduced by 172 kgCO_2_/t·steel relative to the use of coke. The use of biochar as a converter heating agent can contribute to the elimination of 330 million tons of scrap through furnace–converter long-process steelmaking, yielding an annual reduction in CO_2_ emissions of 158 million tons.

## 1. Introduction

**Figure 1:**
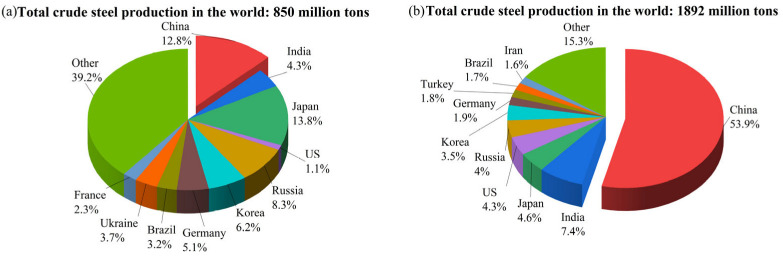
Global crude steel production in (**a**) 2001 and (**b**) 2023 [13].

With the increasing improvement and explosive development of industry in China, domestic crude steel production has grown rapidly since the 20th century; Figure 1 shows the top 10 largest producers of crude steel in 2001 and 2023. Steel production in China has increased from 115 million tons to 1.019 billion tons, and the proportion of crude steel production in the long process of a blast furnace–converter was 90.5% in 2022 according to the World Steel Association.

Metallurgical processes rely heavily on resources such as iron ore and coal, resulting in high energy consumption [1,2]. Consequently, the steel sector has evolved into a major producer of carbon emissions, second only to the power industry. The amounts of CO_2_ emitted for each ton of steel for blast furnace–converter long-process smelting are close to three to four times higher than those for electric arc furnace steelmaking, with emissions of 1.8 to 2.0 tons of CO_2_/t steel [3,4]. However, considering China’s actual situation of low scrap reserves and high iron production, blast furnace–converter long-process smelting may still occupy a dominant position in the industry over the next 50 years [5]. Although carbon capture and storage can reduce the net emission of CO_2_, it increases the cost; thus, reducing carbon emissions from the steelmaking source is the key to realizing the goal of carbon neutrality [6]. Scrap is a growing renewable resource, and recycling scrap in the metallurgical industry, where carbon emissions are under great pressure, is an important strategy for energy conservation and environmental protection [7,8,9,10]. High-scrap-ratio converter smelting can not only consume China’s scrap resources but can also reduce carbon emissions from the blast furnace–converter long-flow process [11]. Therefore, high-scrap-ratio smelting in converters is essential and represents a significant direction for the future evolution of converter smelting [12].

The converter methods used to improve the scrap ratio mainly include secondary combustion, adding a heating agent to the furnace mouth, preheating the scrap, and using a lid to keep the molten iron tank warm [14,15,16,17,18]. In China, when a converter is used for smelting with a high scrap ratio, a charcoal heating agent is most commonly used as a supplementary source of converter heat, as this method provides simple operation and a mature application. Charcoal heating agents mainly comprise coke or pulverized coal, and their exothermic process in the converter is a typical fossil fuel combustion process, which releases a large amount of CO_2_ while adding heat to the converter [19].

**Figure 2:**
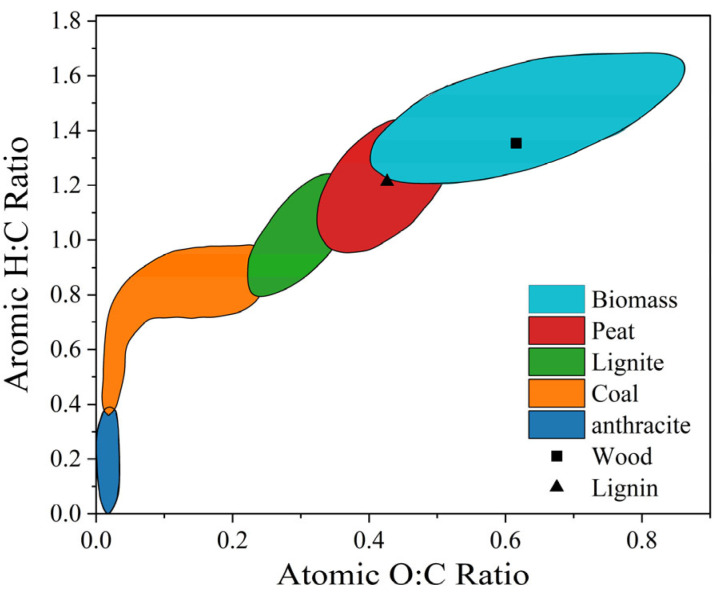
Ven’s diagram of various solid fuels [21].

The steel production industry constitutes about 20% of the total energy consumption across all industries [20]. At the same time, the iron and steel industry requires intensive fossil fuel consumption for different processes, resulting in significant CO_2_ emissions. The use of biomass fuels in the steelmaking process can substantially cut down the carbon emissions of the steel production industry [4]. Figure 2 [21] shows the O:C and H:C ratios of various solid fuels; anthracite has the highest O:C and lowest H:C ratios. Biomass has higher O and H contents relative to C; however, because carbon–oxygen and carbon–hydrogen bonds are less energetic than carbon bonds, the energy released from the combustion of biomass is low. Therefore, raw biomass cannot be used directly for metallurgical applications. In addition, the transportation process for biomass is expensive, and it requires pretreatment and modification processes. Table 1 lists the predominant biomass treatment methods currently available.

In the metallurgical industry, biomass is primarily processed into biochar through pyrolysis [33]. Replacing up to 60% of the pulverized coal in the sintering process with biochar can ensure the quality of the sintered ore and reduce the emissions of NOx [34,35]. Owing to its high reactivity, low density, and good abradability, biochar can be mixed with pulverized coal for use as a blowing fuel in converters. Using biomass charcoal to replace 10% of the bituminous coal during blasting can reduce CO_2_ emissions by approximately 25 kg for every 1 t of iron produced [36,37]. Based on its reduction characteristics, biomass charcoal can be used as a reducing agent in the production of pelletized ores. When the biomass is added at 20% and roasted for 7.5 min at a roasting temperature of 800 °C, an iron ore concentrate that achieves a total iron content of 71.07% and a recovery efficiency of 94.17% is attainable [38]. Research has shown that using rice husks or coconut shells as the raw material for the production of biomass coke can yield an apparent density of up to 1.414 g/cm^3^ and maximum compressive strength of 95.11 MPa at room temperature; therefore, this material can be used as a substitute for part of the coke used in blast furnace ironmaking [39]. In steelmaking, biochar can be used as a foaming agent in electric furnace smelting because of its high volatile content after pyrolysis, which provides heat and maintains the reaction in the slag phase for a longer period of time [40,41]. When the main raw material is molten iron, there is plenty of heat in the smelting process; however, under the condition of a high scrap ratio, incorporating a heating agent is essential for maintaining thermal equilibrium. The application of biochar in the converter smelting process has been scarcely explored in the published research.

Because the basic industrial market in China is dominated by long-process steelmaking, reducing carbon emissions from converter steelmaking represents an important component of China’s efforts to achieve the temperature goal of the Paris Agreement [42]. Up to now, the primary emphasis of studies has been on examining how energy usage affects CO_2_ emissions in blast furnaces and pre-iron processes [43,44]; less research has been conducted on the carbon emissions of converter processes. Feng et al. (2022) studied the effect of double-blow CO_2_ smelting on carbon emissions in a converter and found that using the top and bottom double-blow converter CO_2_ smelting process reduced carbon emissions by 23.8 kg per ton of steel [45]. Utilizing a high proportion of scrap in converter smelting can decrease the converter’s carbon emissions to a certain extent if biochar is used as a heating agent to further improve the converter scrap ratio of the converter, which will help further reduce carbon emissions [46].

This research assesses the carbon dioxide emissions from the converter process through an LCA approach. Not only are the direct carbon emissions of the process considered, but the CO_2_ emissions generated by the upstream processes that provide energy and materials for the converter process, as well as the CO_2_ emission offsets of the main products of the converter process also being taken into account. First, the boundaries of the material output and input of the converter process are defined, and the CO_2_ emission factors of various materials and energy media are then calculated. The effects of the converter process’s carbon emissions with different scrap ratios are investigated. Furthermore, the research examines the carbon emissions from the converter process utilizing high scrap rates and biomass charcoal/coke as a heating agent. The results show a progressive decrease in the converter process’s carbon emissions as the scrap ratio increases. Compared with using coke as a converter heating agent, the use of biochar not only increases the scrap ratio at the lowest carbon emissions, but also reduces the CO_2_ emissions. The results of this study suggest that high-scrap-ratio smelting in converters can serve as an effective method to consume scrap in China. Moreover, the use of biochar as a heating agent can facilitate the reduction in carbon emissions in the steel production industry.

## 2. Methodology and Analysis of Carbon Dioxide Emissions

The determination of carbon emissions in the converter process primarily hinges on the materials and energy expended during the smelting operation; however, it is also affected by the calculation method, calculation boundary, and carbon emission factor. The carbon emissions calculated under uniform smelting conditions using different calculation methods can vary significantly. The detailed computational procedure is outlined below: the (i) selection of calculation methods, (ii) determination of calculation system boundaries, (iii) determination and assessment of CO_2_ emission factor coefficients, and (iv) calculation of carbon dioxide emissions [47].

### 2.1. Methodology for Estimating Carbon Dioxide Emissions Within China’s Steel Sector

Based on the energy consumption per ton of steel in the iron and steel industry [48], three main methods can be used to calculate the CO_2_ emissions from China’s steel sector: (1) direct CO_2_ emissions, (2) CO_2_ emissions resulting from energy utilization, and (3) CO_2_ emissions based on LCA theory.

#### 2.1.1. Direct CO_2_ Emissions

Direct CO_2_ emissions from steel companies are calculated as the amount of CO_2_ emitted to the atmosphere within the defined boundary of the emitting company. This method is mainly based on the calculation method for carbon emissions from steel enterprises proposed by the United Nations Intergovernmental Panel on Climate Change (IPCC). The carbon input encompasses CO_2_ emissions resulting from the utilization of fossil fuels, carbonaceous melts, and other raw materials rich in carbon. Conversely, the carbon output is primarily constituted by the CO_2_ emission reductions achieved through various carbon-based products.

#### 2.1.2. CO_2_ Emissions from Energy Consumption

CO_2_ emissions from energy consumption refer to the CO_2_ generated by energy media in the production process. And the carbon emissions of the production process are calculated based on the amount of an energy medium used within the boundary. This calculation method for carbon emissions is based on the voluntary action plan of the Japan Iron and Steel Federation (JISF) and the carbon emissions calculated by refining the system boundary. The carbon inputs mainly include direct carbon emissions due to the use of fossil fuels and indirect carbon emissions from energy used upstream; the carbon outputs mainly include CO_2_ emission credits from energy-containing products, by-products, and power media.

#### 2.1.3. Life Cycle Assessment (LCA)-Based CO_2_ Emissions

The LCA-based CO_2_ emission calculation method is based on a synthesis of the two versions of the World Steel Association (WSA) CO_2_ emissions calculation method. LCA-based CO_2_ emissions are highly operable and suitable for China’s steel production industry. The carbon inputs include both the direct carbon emissions of the enterprise and the CO_2_ emissions generated by the upstream enterprises that provide energy resources to the enterprise; the carbon outputs include the CO_2_ offsets of the enterprise’s output products, by-products, etc.

### 2.2. Determination of CO_2_ Emissions from Converter Steelmaking Operation

This study calculates the carbon emissions from the converter smelting process by referring to the LCA-based carbon emission calculation method for steel companies. As this calculation method considers all materials and energy media, it facilitates side-by-side comparisons between different converter smelting operations. The LCA-based process CO_2_ emissions are calculated as the sum of the direct carbon emissions and indirect carbon emissions minus any carbon credits. The direct emissions are calculated by converting the [C] content of the carbonaceous feedstock to CO_2_ emissions. Indirect carbon emissions are mainly CO_2_ emissions from the energy resources used in the production of the previous process and its collection and transportation. However, the CO_2_ emissions in the collection and transportation processes are not easy to determine and are thus ignored in this calculation. Carbon offsets are the CO_2_ emissions offset by the products produced in the converter process; for example, when slag is sold as cement, the carbon emissions should be reduced by 44% relative to ordinary cement production. The equation for calculating CO_2_ emissions is as follows:

(1) $$E_{CO_{2}}^{BOF}=\sum_{i=1}^{m}(EF_{i}+EF_{i}^{\prime })\times F_{i}+\sum_{j=1}^{n}(EF_{j}+EF_{j}^{\prime })\times F_{j}-\sum_{k=1}^{l}(EF_{k}+EF_{k}^{\prime })\times F_{k}$$

where $E_{CO_{2}}^{BOF}$ is the process carbon emissions from the converter based on LCA [tCO_2_/t steel], $EF_{i}$ is the carbon emission factor for direct carbon emissions from the converter feedstock [tCO_2_/t, tCO_2_/km^3^], $EF_{i}^{\prime }$ is the carbon emission factor for indirect carbon emissions from the converter feedstock [tCO_2_/t, tCO_2_/km^3^], $F_{i}$ is the amount of raw material fed into the converter process [t, km^3^], $EF_{j}$ is the carbon emission factor for direct carbon emissions from auxiliary energy media [tCO_2_/tce, tCO_2_/t, tCO_2_/km^3^], $EF_{j}^{\prime }$ is the carbon emission factor for indirect carbon emissions from auxiliary power media [tCO_2_/tce, tCO_2_/t, tCO_2_/km^3^], $F_{j}$ is the amount of the auxiliary energy medium fed into the converter process [tce, t, km^3^], $EF_{k}$ is the direct carbon emission factor for product carbon credits [tCO_2_/t, tCO_2_/km^3^], $EF_{k}^{\prime }$ is the indirect carbon emission factor for product carbon credits [tCO_2_/t, tCO_2_/km^3^], $F_{k}$ is the amount of products and by-products of the converter process [t, km^3^], and m, n, and l indicate types of raw materials, power media, and products and by-products, respectively.

### 2.3. System Boundary and Parameterization

**Figure 3:**
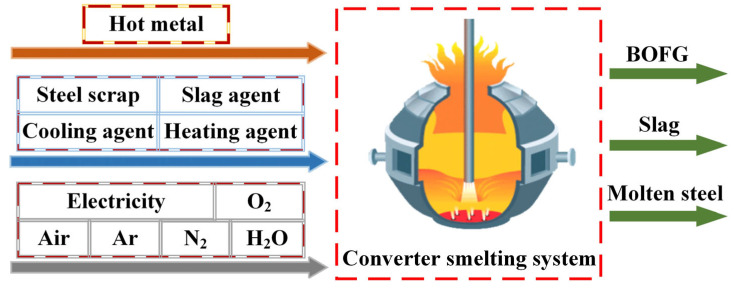
Converter smelting system boundary.

The boundary of this research is about the converter steelmaking process (BOF). The substances input to the BOF boundary include the hot metal, steel scrap, slag agent, cooling agent, power medium, and heating agent, whereas the substances output from the BOF boundary include finished molten steel, converter gas (calorific value recovery), converter slag, and steel. Figure 3 shows a schematic of the converter smelting boundary.

### 2.4. Determination of CO_2_ Emission Factors

CO_2_ emission factors are key parameters in the calculation of CO_2_ emissions. They represent reference standards for carbon dioxide emissions per unit of fossil fuels, power media, products, etc. According to the definition in the calculation methodology above, carbon emission factors are categorized into direct and indirect emission factors.

This research adopts two methodologies for calculating the WSA: the Chinese standards GB/T 34194-2017 [49] for assessing energy efficiency in converter operations, which was established in 2017, and GB/T 2589-2020 [50] for the general principles of comprehensive energy consumption calculation, introduced in 2020. Furthermore, the study also considers the ISO 14404-1 [51] approach for determining the carbon dioxide emission intensity in the iron and steel industry, specifically for converter steelmaking. Consequently, the CO_2_ emission factors are ascertained by integrating the actual operational data from the converter process.

#### 2.4.1. Determination of Direct CO_2_ Emission Factors

Direct CO_2_ emissions in the converter process mainly originate from carbon-containing materials entering and leaving the converter process boundary, including energy sources, fluxes, and heating agents that contain the element [C]. The direct CO_2_ emission factor is obtained mainly from the CO_2_ converted from [C] in the material. If a company is unable to measure the [C] content of the raw material, the default value in the WSA calculation method can be used instead.

In terms of iron and steel materials, hot metal is the main raw material for converter steelmaking, and the [C] content of molten iron is between 4.1% and 4.7% during industrial tests. Thus, the weighted average of the [C] content in molten iron is 4.48%, and the direct emission factor for hot metal is calculated as 0.1644 tCO_2_/t·iron based on the [C] content of molten iron. To avoid repeated carbon dioxide emission calculations at the enterprise level in the steel production industry, carbon emissions from the production of steel scrap are not considered. Moreover, the [C] content of steel scrap is very small; therefore, the default value of the direct emission factor for steel scrap provided by the WSA is 0.0128 tCO_2_/t.

Flux is an important auxiliary material that must be used in the smelting process of a converter; fluxes can be divided into slag, cooling, and heating agents according to their use. The addition of a slag agent is conducive to the removal of impurities in molten steel and the protection of the furnace lining. Cooling agents are primarily used to maintain the heat balance of the converter and promote slagging. Heating agents are primarily used to supplement the heat of the converter smelting. The types of slag agents used in the converter smelting process include limestone, lime, dolomite, and calcined dolomite.

Limestone and dolomite are obtained by mining and crushing, and their direct emission factors have default values of 0.44 tCO_2_/t and 0.471 tCO_2_/t, respectively. Lime and calcined dolomite, which are obtained by processing limestone and dolomite, respectively, have negligible [C] contents; thus, after being added to the converter, their direct emission factors are recorded as zero. Similarly, sintered ores, which are used as cooling agents, contain no elemental [C]; therefore, their direct emission factor is also zero.

Converter heating agents mainly include coke and biochar, and the direct emission factor of coke is obtained by the conversion of the [C] according to its dry basis composition, yielding 3.04 tCO_2_/t. According to LCA theory, biomass can be regarded as solar energy stored in the chemical bonds of carbohydrates through the photosynthesis process, and the CO_2_ emitted to the atmosphere from its combustion conversion can be regarded as equal to the CO_2_ absorbed in its previous growth; therefore, the direct emission factor for biochar is zero [52].

The power media used in the converter include electricity, oxygen, and nitrogen; the direct carbon emission factors for these media are zero because they do not contain [C]. The products of the converter mainly include steel, converter gas, and slag; the [C] content in steel is approximately 0.045%, yielding a direct emission factor of 0.0016 tCO_2_/t, whereas the direct emission factor of steel slag is 0. The converter gas captured during the test period contained 53.30% CO and 17.74% CO_2_, yielding a direct emission factor of 1.395 tCO_2_/km^3^.

**Figure 4:**
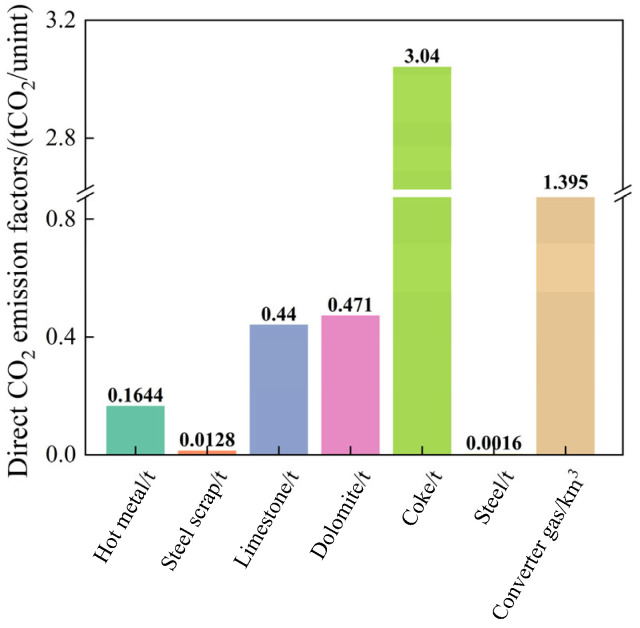
Distribution of direct carbon emission factors.

The direct emission factors for each raw and auxiliary material and power medium are shown in Figure 4.

#### 2.4.2. Determination of Indirect CO_2_ Emission Factors

The direct carbon emissions of materials used in the converter cannot reflect the impact of different raw materials, iron composition, and power media used in the converter on the carbon dioxide emissions of long-distance steel production. It is imperative to factor in the CO_2_ emissions from the converter’s raw materials throughout the manufacturing process. The coefficients reflecting the carbon emissions generated per unit mass of raw material in production are the indirect emission factors. The determination process is described below.

The indirect emission factor of the hot metal is strongly influenced by the operational status of the blast furnace steelmaking procedure; therefore, the default value of the indirect emission factor for pig iron given by the WSA is used in this calculation: 1.855 tCO_2_/t.

The electricity used in steel mills mainly originates from self-generated electricity and purchased electricity, both of which use different powers. For the convenience of different smelting programs and comparisons with other enterprises, the carbon emission factor of electricity is considered as the carbon emissions during power generation in different regions. The emission factor for electricity in North China is quantified at 0.9419 tCO_2_ per MW·h. The indirect emission factors for oxygen, nitrogen, argon, water, and air are determined according to the ratio of their energy consumption coefficients to that of electricity. The results of the calculations are the same as those reported by Feng et al. [45]. The steam pressure of the plant in the study is 1.0 MPa and the discounted standard coal factor is 0.0949 kgce/t; thus, the indirect emission factor is 0.7273 tCO_2_/t.

The WSA assigns standard emission factors for the indirect emissions from limestone crushing and dolomite processing, which are 0.003 and 0.062 tCO_2_/t, respectively. The indirect emission factors for lime and calcined dolomite are derived from the consumption of limestone and dolomite in their production, amounting to 1.035 and 1.1 tCO_2_/t, respectively. The sinter does not contain [C] but generates carbon emissions during production. The indirect emission factor for the sinter has a default value of 0.329 tCO_2_/t in the WSA. The indirect carbon emission factor for the coke heating agent also has a default value in the WSA of 0.463 tCO_2_/t.

**Figure 5:**
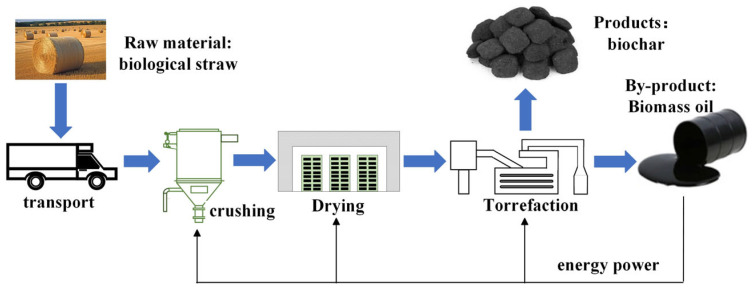
Biomass charcoal production process.

**Figure 6:**
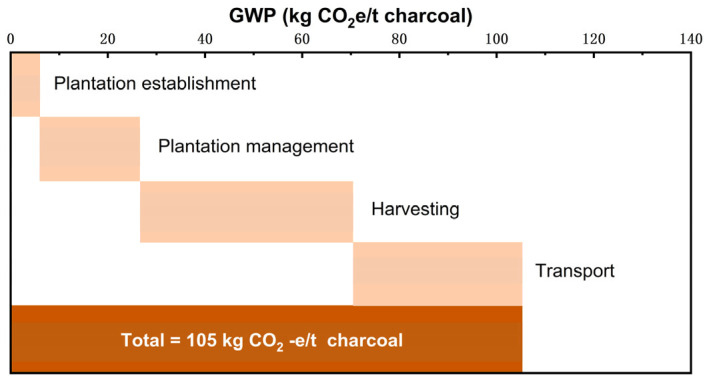
Composition of carbon emission factors for biomass charcoal [53].

A flowchart of the production of biomass char is shown in Figure 5. The biomass pyrolysis process, to obtain biochar, also produces biomass oil, combustible gasses, and water vapor. Accordingly, the carbon emissions generated in the process of the transportation and production of biomass charcoal are 105 kgCO_2_/t, and thus the indirect emission factor is 0.105 tCO_2_/t. The specific composition of the indirect carbon emission factors is shown in Figure 6 [53].

**Figure 7:**
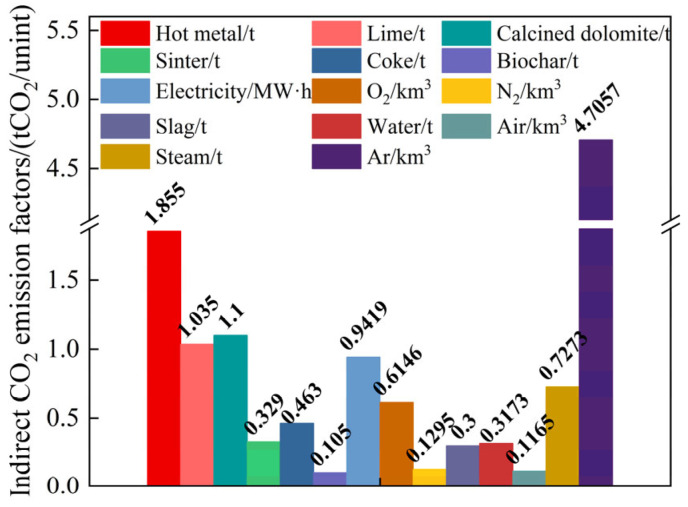
Distribution of indirect carbon emission factors.

The by-products of the converter smelting output mainly include converter gas and slag. The carbon emission factor for the converter gas was calculated previously. Although slag does not contain [C], it can offset some of the carbon emissions when used as a raw material for cement; the average carbon dioxide emission offset factor for cement production from converter slag is 0.3 tCO_2_/t. The indirect emission factors for each raw and auxiliary material and energy medium are shown in Figure 7. Table 2 lists the direct and indirect emission factors for the raw and auxiliary materials, power media, and products required for smelting in the converter.

## 3. Calculation and Assessment of CO_2_ Emissions for Converter Smelting Processes

### 3.1. Industrial Applications of Converter Smelting with Different Scrap Ratios

In this study, industrial tests were conducted on a 300 t converter with varying amounts of scrap per furnace; a total of eight smelting schemes were designed. Smelting scheme 1 (SH1) had a scrap dosage of 0–10 t, and each subsequent test scheme increased the scrap dosage by 10 t up to 80 t (SH8). The scrap ratio of each smelting scheme was obtained by averaging the scrap ratios of each furnace at the time of the test, and the actual scrap dosage data during the smelting period of the converter are listed in Table 3.

During the test period, the composition of the molten iron was relatively stable: the [C] content of the hot metal remained between 4.1% and 4.7%, and the [Si] content of the hot metal stabilized between 0.2% and 0.5%. The temperature of the molten iron remained in the range of 1280–1340 °C, and the smelting process stabilized without double-slag operation.

### 3.2. Modeling the Utilization of the Converter Heating Agent

The utilization rate of charcoal heating agents shows a trend of decreasing with the increase in the scrap ratio, and the combustion of the heating agent will emit a large amount of CO_2_, which markedly affects the CO_2_ emissions of the converter process. Therefore, it is necessary to determine the utilization coefficient of the charcoal heating agent with high scrap ratios.

**Figure 8:**
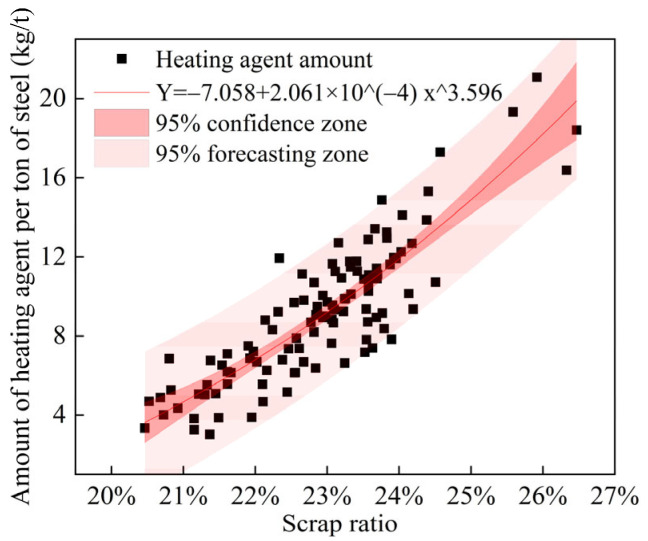
Coke usage vs. scrap ratio during test period.

The heat balance of the converter is mainly related to the physical and chemical heat of the incoming raw materials, and the control of the composition of the end temperature. Smelting furnaces with [C] contents of iron in the range of 4.2–4.4% and [Si] contents in the range of 0.3–0.5% are selected; the furnaces do not use cooling agents, limestone, or dolomite in the melting fluxes. Therefore, the obtained heat balance of the screened furnaces is mainly related to the scrap ratio and the amount of heating agent. A scatter plot of the variation in the amount of heating agent with the scrap ratio is shown in Figure 8. Curve fitting is performed on the data, and the following power equation fits well (R^2^ > 0.75):

(2) $$Y=-7.058+2.061\times 10^{-4}x^{3.596}$$

where Y is the amount of steel coke used [kg/t·steel], and x is the scrap ratio [%].

The derivation of the above equation yields the following equation for the variation in coke dosage with the scrap ratio:

(3) $$\Delta Y=7.411\times 10^{-4}x^{2.596}$$

where ΔY is the coke usage per ton of steel at a scrap ratio x with a further 1% increase in the scrap ratio [kg/t·steel].

By establishing the material and heat balance model of the converter, it is calculated that for every 1% increase in the scrap ratio, a ton of steel needs to compensate for a heat of 18694.05 kJ. Because coke has a low calorific value of 32768.70 kJ/kg, for every 1% increase in the scrap ratio, a ton of steel requires 0.57 kg of coke. Given the requirement for the use of a charcoal heating agent, and the actual amount of coke required for each increase of 1% in the scrap ratio, the coefficient of coke utilization can be obtained as follows:

(4) $$\eta =7.698\times 10^{2}x^{-2.596}$$

where η is the coke utilization factor [%].

In this study, scrap ratios of more than 20% are considered high-scrap-ratio smelting conditions. For this study, high scrap ratios varying from 20% to 40% are considered at scrap ratio increments of 2%; thus, a total of 11 high-scrap-ratio smelting modes are calculated. As the proportion of scrap rises, the consumption of heating agents and oxygen also escalates. Table 4 details the oxygen consumption per ton of steel and the quantities of carbon heating agents utilized across various smelting methods.

### 3.3. CO_2_ Emission Analysis for Smelting with Different Scrap Ratios

**Figure 9:**
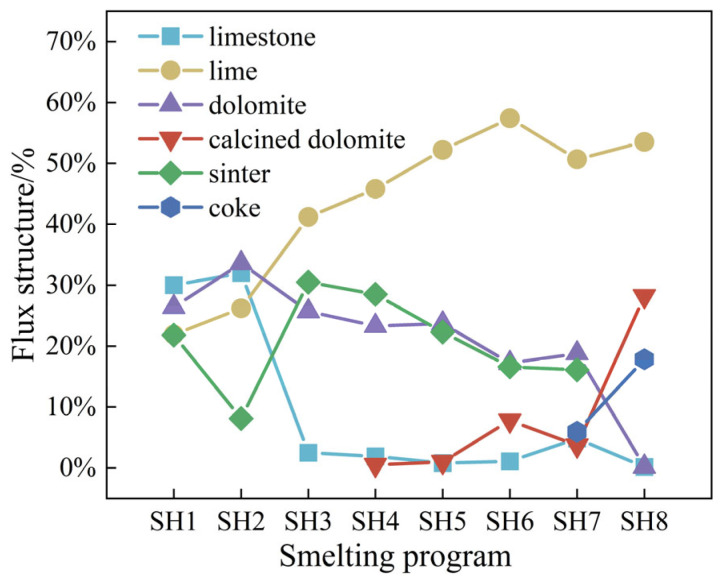
Variation in flux dosage with scrap ratio.

Figure 9 illustrates the variations in the flux structure throughout the smelting process with varying scrap ratios. From SH1 to SH8, the total proportion of sinter and limestone in the melt decreases as the scrap ratio increases. This is mainly because the total heat of the melt pool decreases as the scrap ratio increases. Therefore, the amount of sinter used as a cooling agent should theoretically decrease. Moreover, the limestone, as a slag agent, will absorb heat by decomposition rather than through the use of lime, and thus the limestone content will decrease with the increasing scrap ratio. After SH4, as the scrap ratio continues to increase, calcined dolomite gradually begins to be used as a slag agent in place of dolomite to maintain sufficient heat of the melt pool. Moreover, coke is used as a heating agent in SH7 and SH8. As the scrap ratio increases from 22.14% to 24.21%, the proportion of coke reaches 18%, and no cooling agent is used. Thus, the slag agent consists of only calcined dolomite and lime, which may indicate that the effective heat utilization of the heating agent is negatively correlated with the increase in the scrap ratio. Overall, from SH1 to SH8, the specific change in flux structure is that the flux with a cooling effect is gradually reduced, and the heating agent is gradually introduced.

**Figure 10:**
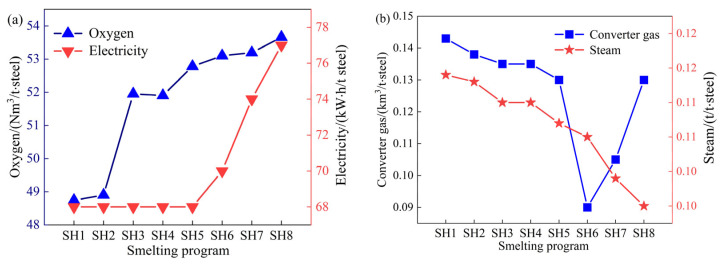
Variations in the (**a**) energy media and (**b**) by-products with the scrap ratio.

The consumption of oxygen and electricity in the power medium of the converter is affected by the scrap ratio, as shown in Figure 10. The consumption of both oxygen and electricity rises as the scrap ratio increases. Among the by-products of converter smelting, the amount of converter gas decreases as the scrap ratio increases before coke is used, the amount of converter gas increases after the addition of a heating agent, and the amount of steam decreases as the scrap ratio increases throughout the tests. The above analysis shows that with the increase in converter scrap, the energy medium used in converter smelting increases, but the amount of energy medium produced in smelting decreases. After the converter smelting uses coke as the heating agent, although the amount of converter gas recovery increases, the overall energy consumption should also be increased.

**Figure 11:**
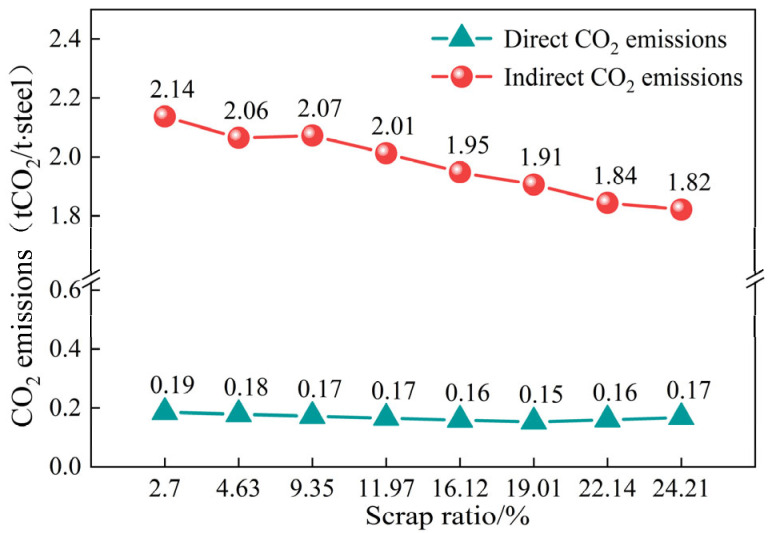
Variation in direct and indirect carbon emissions with the scrap ratio.

As shown in Figure 11, the direct CO_2_ emissions from the converter smelting process decrease gradually as the scrap ratio increases, while the direct carbon emissions increase when coke is used. The indirect CO_2_ emissions from the converter process also decrease gradually as the scrap ratio increases; however, the decreasing trend is significantly lower when coke is used in SH7 and SH8. According to the trend shown in Figure 11, direct carbon emissions from the converter tend to increase if it relies only on the use of coke as a heating agent.

**Figure 12:**
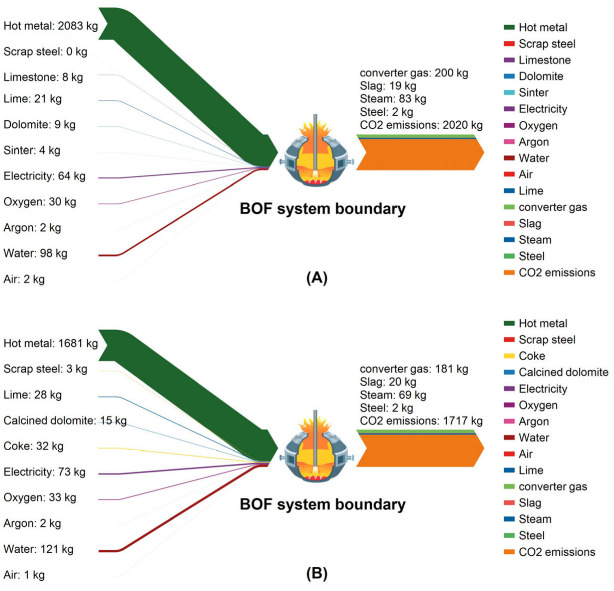
Analyses of CO_2_ emissions of the converter process ((**A**): CO_2_ emission distribution with a scrap ratio of 2.7%; (**B**): CO_2_ emission distribution with a scrap ratio of 24.21%).

During the trials, the CO_2_ emissions from the converter operation progressively diminished as the scrap ratio increased; Figure 12 shows the Sanki diagrams of the CO_2_ emissions of the converter process for scrap ratios of 2.70% and 24.21%. The converter process’s carbon emissions are predominantly influenced by the hot metal; increasing the scrap ratio and decreasing the hot metal in the raw material are the main reasons for the decrease in the carbon emissions of the converter process. In terms of carbon input, in addition to molten iron, the use of coke as a heating agent at high scrap ratios increases the oxygen consumption, electricity consumption, and water usage, and the carbon emissions caused by these power media increase. In terms of product carbon credits, a higher proportion of scrap material leads to a decrease in the recovery of converter gas and steam, and the total amount of carbon credits decreases. Overall, from SH1 to SH8, for every 1% increase in the converter scrap ratio, the converter process reduces by 14.09 kgCO_2_/t·steel.

### 3.4. CO_2_ Emissions Analysis for High-Scrap-Ratio Smelting

**Figure 13:**
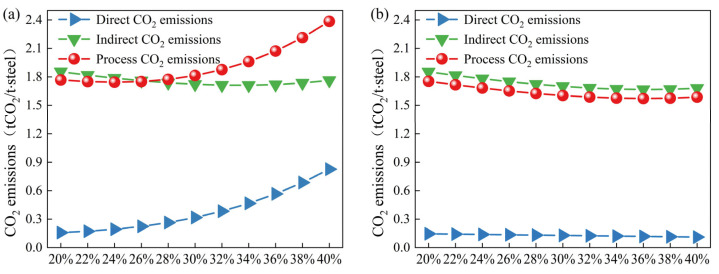
Carbon emissions of the converter process with high scrap ratios ((**a**): coke as the heating agent; (**b**): biochar as the heating agent).

The CO_2_ emissions under high scrap ratios are shown in Figure 13; Figure 13a,b show the variation in carbon emissions with the scrap ratio when using coke and biochar as heating agents, respectively. As shown in Figure 13, the direct CO_2_ emissions gradually increase as the scrap ratio increases when coke is used as the heating agent, whereas the direct emissions decrease when biochar is used as the heating agent. The indirect CO_2_ emissions of the converter process initially decrease and then increase as the scrap ratio increases, and the total process CO_2_ emissions also decrease and then increase.

Compared with the use of coke as the heating agent for the converter, the reduction in CO_2_ emissions when using biochar is mainly due to the reduction in direct CO_2_ emissions. When the scrap ratio reaches 40%, the direct CO_2_ emissions with use of coke as the heating agent are 0.827 tCO_2_/t·steel, and the direct carbon emissions with the use of biochar are only 0.111 tCO_2_/t·steel; therefore, using biochar can effectively reduce the CO_2_ emissions of the converter. In addition, the calculation results indicate that when coke is used as the heating agent, the carbon emissions of the converter process are the lowest (1.743 tCO_2_/t steel) for a scrap ratio of 24%; when biochar is used as a heating agent, the carbon emissions of the converter process are the lowest (1.571 tCO_2_/t steel) for a scrap ratio of 36%. In conclusion, using biomass charcoal as a converter heating agent can reduce the converter emissions by 172 kgCO_2_/t steel and can effectively improve the scrap ratio, which is conducive to solving the problem of scrap consumption and treatment in China.

**Figure 14:**
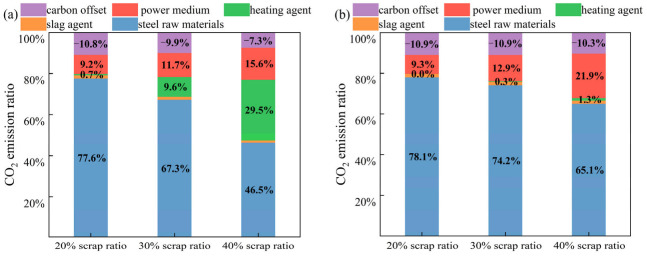
Carbon emissions distribution in the converter process with high scrap ratios ((**a**): coke as the heating agent; (**b**): biochar as the heating agent).

Figure 14 shows the distribution of CO_2_ emissions when two types of heating agents are used: coke and biochar. As depicted in Figure 14a, the carbon emissions attributed to the consumption of hot metal and steel materials progressively diminish as the scrap ratio rises. In contrast, the carbon emissions resulting from the utilization of power media and heating agents steadily increase. Figure 14b shows that the CO_2_ emissions from the power media increase as the scrap ratio increases, whereas the carbon emissions contributed by the heating agent using biochar remain almost constant and account for a small percentage. In addition, the heating agent is considered to be completely combusted into CO_2_. Therefore, the value of carbon credits for recovering both the converter gas and steel slag decreases as the scrap ratio increases. In summary, using coke or biochar as a heating agent in the converter will increase the amount of power medium O_2_ consumption, which will increase the CO_2_ emissions of the converter process.

According to the above results, enhancing the scrap ratio in the smelting process can significantly lower the CO_2_ emissions associated with the converter operation. When there is insufficient heat in the converter smelting process, a charcoal heating agent can be used to supplement the heat for the smelting process; nevertheless, the utilization rate declines as the scrap ratio is raised. Compared with the use of coke as a heating agent, the use of biochar can effectively decrease the CO_2_ emissions of the converter operation. In the steel production industry, where the pressure to reduce carbon emissions is significant, the use of biomass charcoal as supplementary heat can increase the scrap ratio of converter smelting.

## 4. Conclusions

This research introduces a methodology for calculating CO_2_ emissions in converter smelting and establishes the boundary of the converter’s CO_2_ emissions. The direct, indirect, and process carbon emissions of the converter process are analyzed under different scrap ratios. The CO_2_ emissions when coke and biomass charcoal are used as heating agents are calculated under the condition of a high scrap ratio. The main conclusions are summarized as follows:

(1) Based on statistical data for the coke dosage under high scrap ratios, the utilization rate of the charcoal heating agent decreases gradually as the scrap ratio is raised, and the relationship between the utilization rate of the charcoal heating agent and the scrap ratio can be expressed as $\eta =7.698\times 10^{2}x^{-2.596}$.
(2) As the scrap ratio is raised, the dosages of lime and calcined dolomite in the flux increase, while the dosages of limestone and dolomite decrease; the consumption of oxygen and electricity increases, and the amounts of recovered converter gas and steam decrease. The overall carbon emissions from the converter process decrease with the increasing scrap ratio; every 1% increase in the scrap ratio results in a reduction of 14.09 kgCO_2_/t·steel for the converter process.
(3) The use of biochar as a heating agent in converter smelting with a high scrap ratio can effectively increase the scrap ratio of converter smelting and reduce carbon emissions. The use of biochar as a heating agent can increase the scrap ratio to 36%, and the CO_2_ emissions for a ton of steel are 1.571 tCO_2_, which represents a reduction in emissions of 172 kgCO_2_/t·steel compared with the use of coke. The annual output of blast furnace–converter long-process steelmaking in China is approximately 917 million tons; therefore, up to 330 million tons of scrap can be consumed, yielding a reduction in CO_2_ emissions of 158 million tons.

In this study, the CO_2_ emission factors of the converter carbon emission boundary, all materials, and power media were investigated, and the effects of smelting with different scrap ratios on the carbon emissions of the converter were analyzed. The research calculated how CO_2_ emissions fluctuate as the scrap ratio in the converter increases, considering the use of charcoal as a heating agent. The findings indicate that the converter process’s total CO_2_ emissions progressively diminish as the scrap ratio is raised. When coke is used as a heating agent, the CO_2_ emissions of the converter increase with an increase in the scrap ratio. When biochar is used as a heating agent, the CO_2_ emissions decrease gradually with an increase in the scrap ratio of the converter. However, the utilization rate of the charcoal heating agent in the converter is gradually reduced as the scrap ratio is raised. Therefore, improving the scrap ratio of the converter should be achieved through secondary combustion technology, scrap preheating technology, iron ladle cover technology, etc., to ensure that the thermal efficiency of the fuel is maximized. This further increase in the scrap ratio of the converter steelmaking process is conducive for reducing carbon emissions. Combined with the contribution of biochar to the reduction in carbon emissions in converter smelting calculated in this study, biochar can be used as a heating agent in future converter smelting with low energy consumption, which can increase the scrap ratio and reduce smelting carbon emissions. This study serves as a supplement to smelting technology with a high scrap ratio in the converter process. The effects of using coke and biochar as heating agents on CO_2_ emissions in the converter process are analyzed, with the aim of providing guidance and references for the reduction in CO_2_ emissions in long-process smelting.

## Figures and Tables

**Table 1:** Major treatment methods for biomass utilization.

Treatment	Raw Material	Process Method	Main Products	Ref.
Thermochemical treatment	Lignocellulosic biomass	Combustion, gasification, pyrolysis, and liquefaction	Biomass charcoal, biomass oil	[22,23,24]
Chemical transformation	Low lignocellulosic biomass	Hydrolysis, organic melt dissolution, ionic liquid pretreatment	Chemicals such as acetic acid, polysaccharides	[25,26,27]
Physicochemical transformation	Vegetable oils, animal fats	Ester exchange and esterification reactions	Biodiesel	[28,29,30]
Biological treatment	Biomass, microorganisms, and enzymes	Biodegradation	Biofuels, aldehydes, and phenolic acids	[31,32]

**Table 2:** Carbon emission factors.

Project	Units (Unit)	Direct Emission Factor (tCO_2_/Unit)	Indirect Emission Factor (tCO_2_/Unit)
Hot metal	t	0.1644	1.8550
Steel scrap	t	0.0128	0
Limestone	t	0.4400	0.0030
Lime	t	0	1.0350
Dolomite	t	0.4710	0.0620
Calcined dolomite	t	0	1.1000
Sinter	t	0	0.3290
Coke	t	3.0400	0.4630
Biochar	t	0	0.1050
Electricity	MW-h	0	0.9419
O2	km^3^	0	0.6146
N2	km^3^	0	0.1295
Ar	km^3^	0	4.7057
Water	t	0	0.3173
Air	km^3^	0	0.1165
Steel	t	0.0016	0
Slag	t	0	0.3000
Converter gas	km^3^	1.3954	0
Steam	t	0	0.7273

**Table 3:** Industrial test program for converter smelting with different scrap ratios.

Smelting Program	Scrap Ratio/%	Scrap Usage/t	Data Volume
SH1	2.70	0~10	1806
SH2	4.63	10~20	92
SH3	9.35	20~30	954
SH4	11.97	30~40	701
SH5	16.12	40~50	618
SH6	19.01	50~60	426
SH7	22.14	60~70	2413
SH8	24.21	70~80	488

**Table 4:** Heating agent and oxygen consumption in high-scrap-ratio smelting (per ton of steel).

Project	20%	22%	24%	26%	28%	30%	32%	34%	36%	38%	40%
heating agent/kg	4.24	9.93	18.08	29.16	43.69	62.22	85.32	113.56	147.58	188.00	235.50
utilization factor/%	32.28	25.20	20.11	16.33	13.48	11.27	9.53	8.14	7.02	6.10	5.34
oxygen/Nm^3^	55.61	63.83	76.10	93.23	116.07	145.53	182.52	228.04	283.11	348.77	426.13

## Data Availability

The data that were used are confidential.

## Glossary

LCA	Life cycle assessment
IPCC	United Nations Intergovernmental Panel on Climate Change
JlSF	The Japan Iron and Steel Federation
WSA	World Steel Association
BOF	Basic oxygen furnace
BOFG	Converter gas
CA	Compressed air
SH1~8	Smelting scheme 1~8
